# Molecular surveillance of spotted fever group rickettsioses in wildlife and detection of *Rickettsia sibirica* in a Topi (*Damaliscus lunatus* ssp. *jimela*) in Kenya

**DOI:** 10.4102/ojvr.v84i1.1265

**Published:** 2017-01-30

**Authors:** David Ndeereh, Andrew Thaiyah, Gerald Muchemi, Antoinette A. Miyunga

**Affiliations:** 1 Department of Veterinary Services, Kenya Wildlife Service, Kenya; 2 Department of Clinical Studies, University of Nairobi, Kenya; 3 Department of Public Health, Pharmacology and Toxicology, University of Nairobi, Kenya; 4 Forensics and Genetics Laboratory, Kenya Wildlife Service, Kenya

## Abstract

Spotted fever group rickettsioses are a group of tick-borne zoonotic diseases caused by intracellular bacteria of the genus *Rickettsia*. The diseases are widely reported amongst international travellers returning from most sub-Saharan Africa with fever, yet their importance in local populations largely remains unknown. Although this has started to change and recently there have been increasing reports of the diseases in livestock, ticks and humans in Kenya, they have not been investigated in wildlife. We examined the presence, prevalence and species of *Rickettsia* present in wildlife in two regions of Kenya with a unique human–wildlife–livestock interface. For this purpose, 79 wild animals in Laikipia County and 73 in Maasai Mara National Reserve were sampled. DNA extracted from blood was tested using the polymerase chain reaction (PCR) to amplify the intergenic spacer *rpmE-*tRNA^fMet^ and the citrate synthase-encoding gene *gltA*. Rickettsial DNA was detected in 2 of the 79 (2.5%) animals in Laikipia and 4 of the 73 (5.5%) in Maasai Mara. The PCR-positive amplicons of the *gltA* gene were sequenced to determine the detected *Rickettsia* species. This revealed *Rickettsia sibirica* in a Topi (*Damaliscus lunatus* ssp. *jimela*). This is the first report of spotted fever group rickettsioses in wildlife and the first to report *R. sibirica* in Kenya. The finding demonstrates the potential role of wild animals in the circulation of the diseases.

## Introduction

Spotted fever group (SFG) rickettsioses are a group of tick-borne zoonotic diseases caused by over 20 species of the intracellular bacterium *Rickettsia* (Azad & Beard 1998; Parola, Paddock & Raoult 2005; Raoult & Roux 1997; Todar 2012). They occur worldwide and are associated with diseases in animals and humans (Azad & Beard 1998; Parola et al. 2005) and are diagnosed in some international travellers returning from sub-Saharan Africa (Freedman et al. 2006; Jensenius, Parola & Raoult 2006) including Kenya (Rutherford et al. 2004; Yoshikawa et al. 2005). The clinical manifestations in humans are non-specific and mimic those of other diseases such as malaria and flu-like illnesses (Krauss et al. 2003; Roch et al. 2008). They are transmitted by different species of ixodidae ticks and reservoirs include various species of domestic and wild animals (Cowan 2003; Todar 2012). Because wild animals are hosts of many species of ticks, the diseases can be of particular concern where wildlife shares habitats and other resources with humans and domestic animals (Grootenhuis & Olubayo 1993).

The importance of SFG rickettsioses as causes of illnesses in Kenya is underreported and underappreciated despite being reported amongst foreign travellers who visit game reserves (Richards et al. 2010; Rutherford et al. 2004; Yoshikawa et al. 2005). This may be attributed to lack of awareness and the challenges of making diagnoses in febrile patients in Africa (Ari et al. 2011; Brah et al. 2015). The diseases may therefore be amongst the ‘fevers of unknown origin’ whose aetiologies are often not the focus of health providers or are difficult to diagnose because of lack of resources (Brah et al. 2015). Nevertheless, there have been increasing reports in Kenya of SFG rickettsioses in humans, domestic animals and ticks (Macaluso et al. 2003; Maina 2012; Mutai et al. 2013; Richards et al. 2010; Rutherford et al. 2004; Yoshikawa et al. 2005) but information about their presence in wildlife is lacking. In a situation where emerging and re-emerging zoonotic infections are on the increase with over 70% originating from wildlife (Jones et al. 2008), it is important that studies extend to include all aspects of disease epidemiology. Wildlife plays an important role of disease epidemiology but is often neglected in surveillance and detection of diseases. Wildlife can be important reservoir hosts of many tick-borne pathogens including SFG rickettsiae, which can be transmitted to domestic animals and humans in areas where wildlife and domestic animals share habitats and other resources. This study investigates SFG rickettsioses at the wildlife–livestock interfaces in Laikipia County and Maasai Mara National Reserve. It evaluates their presence, prevalence and identifies the species of SFG rickettsiae circulating in wildlife.

## Research method and design

### Study areas and sampling procedure

Blood was collected from immobilised wild animals in Laikipia County and Maasai Mara National Reserve between February 2014 and October 2015. Laikipia County is about 9500 km^2^ and is located in the central region of Kenya to the northwest of Mt. Kenya between 0.88N 36.18E and 0.2667S 37.38E. The Maasai Mara National Reserve is approximately 1510 km^2^ between 1.22S 34.75E and 1.75S 35.42E within Narok County in southwestern Kenya along the border with Tanzania. The main human populations in both areas are pastoralists whose livelihoods are dependent on livestock. Other forms of land use include agriculture, commercial ranching, wildlife conservation and ecotourism. The two areas comprise some of the most important areas for biodiversity in Kenya, and they have large populations of free-ranging wildlife, which share habitats and other resources with humans and domestic animals providing a likely interface for disease transmission. The sampling sites in each study area were selected based on high interaction of livestock and wildlife and accessibility to enable darting of the animals. The coordinates of each sampling site were recorded using a Global Positioning System (GPS) (Garmin GPS 12 XL, Garmin Olathe, KS, USA) and entered into a Geographical Information System (GIS) database.

Convenience sampling of the animals was employed because of the difficulties of constructing a sampling frame in wildlife to allow for random sampling. This method allowed for readily available animals of the target species to be sampled. The target species were those most common in the study areas with the high tendency to interact with livestock. These included buffalo (*Syncerus caffer*), zebra (*Equus burchellii*), Grant’s gazelle (*Nanger granti*), common waterbuck (*Kobus ellipsiprymnus* ssp. *ellipsiprymnus*), impala (*Aepyceros melampus*), Topi (*Damaliscus lunatus* ssp. *jimela*), Coke’s hartebeest (*Alcelaphus buselaphus*) and wildebeest (*Connochaetes taurinus*). To facilitate sample collection, the animals were immobilised following the protocols recommended by McKenzie (1993) by experienced personnel to ensure a humane exercise as much as possible. At least 30 mL of blood was collected from each animal by jugular venipuncture into EDTA-coated tubes and split into four aliquots. Each aliquot was labelled with information identifying the sample number, date, location and animal species and stored frozen in liquid nitrogen (-196 °C) until required for processing.

### DNA extraction

Genomic DNA was extracted from preserved EDTA blood samples using the manufacturer’s instructions for the DNeasy^®^ Blood and Tissue Kits (QIAGEN GmbH, Hilden, Germany). However, these instructions were modified slightly in order to optimise the amount of DNA extracted by increasing the amount of blood from 50 µL to 100 µL as recommended by the manufacturer to 200 µL and reducing the amount of AE buffer from 200 µL to 150 µL. Extracted DNA quality was evaluated using the agarose gel electrophoresis protocol in which an aliquot of the extracted DNA was run on 1.2% agarose gel.

### Screening for Rickettsia

Host DNA was screened for evidence of Rickettsia using PCR assay to amplify the intergenic spacer *rpmE*-tRNA^fMet^ and the citrate synthase-encoding gene *gltA* using previously described primer sets shown in Table 1. The amplifications were carried out in a total volume of 25 µL reaction mix containing 1 µL deoxyribonucleotide triphosphates solution (dNTPs), 2.5 µL standard Taq buffer (Biolabs^®^, New England, UK), 1 µL each of reverse and forward primers, 17.25 µL of DNase/RNase-Free^®^ PCR grade water (QIAGEN, Hilden, Germany), 0.25 µL of Taq DNA polymerase (Biolabs^®^, New England, UK) and 2 µL of template DNA. The amplifications were performed in Applied Biosystems Veriti^®^ 96-well thermocycler (Applied Biosystems, California, USA). The PCR conditions were as follows: 3 min initial denaturation at 95 °C, 35 cycles of 30 s denaturation at 95 °C, 30 s primer annealing at temperatures specific for each of the primers (Table 1), one minute extension at 72 °C and a final 10 minute extension at 72 °C. The mixture was then maintained at 4 °C. A negative control using DNase/RNase-Free^®^ PCR grade water (QIAGEN, Hilden, Germany) was included for quality control. The positive control used was DNA extracted from *Rickettsia africae* isolated from a tick in Kenya. The PCR products were visualised using agarose gel electrophoresis to check for the presence of band and size of amplicon.

**TABLE 1:** Primers used for polymerase chain reaction amplification and sequencing of spotted fever group rickettsiae in wildlife.

Target gene	Primer	5’-Primer sequence-3’	Annealing temperature (°C)	Expected product size (bp)	References
*rpmE-*tRNA^fMet^	*rpmE*F	TTCCGGAAATGTAGTAAATCAATC	54	144	Fournier et al. (2004)
	*rpmE*R	TCAGGTTATGAGCCTGACGA	54		
*gltA*	CS1dF	ATGACTAATGGCAATAATAA	47	1254	Jiang et al. (2005)
	CS1273R	CATAACCAGTGTAAAGCTG	47		
	CS1234R	TCTAGGTCTGCTGATTTTTTGTTCA	50		
	Rp CS877F	GGGGGCCTGCTCACGGCGG	50	382	
	Rp CS1258R	ATTGCAAAAAGTACAGTGAACA	50	

### DNA sequencing and analysis

The *gltA*-positive PCR amplicons of, or close to, the expected product sizes were purified using QIAquick^®^ purification kit (QIAGEN, Hilden, Germany) following the manufacturer’s instructions. Sequencing was done by direct cycle sequencing using the ABI PRISM BigDye Terminator V3.1 cycle sequencing kit and the sequences analysed in an ABI310 DNA analyser (Applied Biosystems, California, USA). Traces were assembled and primer regions trimmed using Geneious v 8.1.6 software. Consensus nucleotide sequences were used to query the GenBank database, and the highest similarity was identified by Basic Local Alignment Search Tool (BLASTN) available from the National Center for Biotechnology Information (Bethesda, MD). This was used to assign identity to the recovered species. The study sequences along with those with closest match in GenBank were aligned using MUSCLE (Edgar 2004). The evolutionary history was inferred by using the maximum likelihood method based on the Tamura 3-parameter model (Tamura 1992). The phylogeny tree with the highest log likelihood (-1233.9127) was developed. The percentage of trees in which the associated taxa clustered together was shown next to the branches. Initial tree(s) for the heuristic search were obtained automatically by applying neighbour-joining and BioNJ algorithms to a matrix of pair-wise distances estimated using the maximum composite likelihood (MCL) approach and then selecting the topology with superior log likelihood value. The tree was drawn to scale with branch lengths measured in the number of substitutions per site. The analysis involved 15 nucleotide sequences. Codon positions included were 1st+2nd+3rd+Noncoding. There were a total of 774 positions in the final dataset. Evolutionary analyses were conducted in MEGA6 (Tamura et al. 2013).

## Results

### Rickettsial infection in animals

**FIGURE 1:**
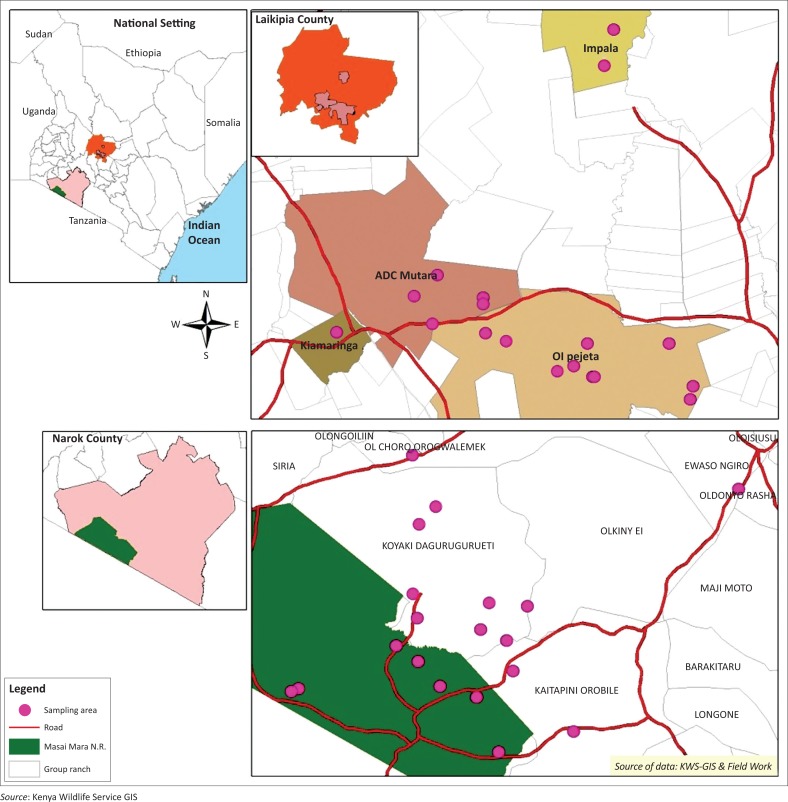
Sampled sites in Laikipia and Maasai Mara.

Animals were sampled in 17 locations in Laikipia County and 19 locations in Maasai Mara National Reserve and the neighbouring areas during six separate expeditions between February 2014 and October 2015 (Table 2). The sampled areas in Laikipia County included nine sites in Ol Pejeta conservancy, five sites in ADC Mutara Ranch and two sites in Mpala Ranch all of which incorporate cattle ranching and wildlife conservation as well as one site in Kiamariga sub-location, which is a community land where free-ranging wildlife interacts with livestock (Figure 1). In Maasai Mara, sampled areas included seven sites inside the National Reserve and 12 sites in neighbouring community group ranches where wildlife interacts freely with livestock (Figure 1).

**TABLE 2:** Animals sampled in each site in both study areas and corresponding dates.

Study areas	Zebra	Buffalo	Common waterbuck	Grant’s gazelle	Topi	Coke’s hartebeest	Wildebeest	Impala
**Laikipia**								
Ol Pejeta Conservancy	27 (0 +) (Jan. 2015)	28 (1 +) (Jan. 2015)	2 (0 +) (Jan. 2015)	**-**	**-**	**-**	**-**	**-**
ADC Mutara Ranch	9 (1 +) (Feb. 2015)	3 (0 +) (Feb. 2015)	**-**	**-**	**-**	**-**	**-**	**-**
Mpala Ranch	**-**	**-**	**-**	7 (0 +) (Feb. 2014)	**-**	**-**	**-**	**-**
Kiamariga	3 (0 +) (Feb. 2015)	**-**	**-**	**-**	**-**	**-**	**-**	**-**
**Maasai Mara**	**-**	**-**	**-**	**-**	**-**	**-**	**-**	**-**
Inside National Reserve	21 (0 +) (July 2014)	**-**	**-**	**-**	10 (3 +)† (July 2015)	5 (0 +) (July 2015)	11 (0 +) (Oct. 2015)	2 (0 +) (Oct. 2015)
Outside National Reserve	**-**	**-**	**-**	**-**	**-**	**-**	24 (1 +) (Oct. 2015)	**-**

† Two herds of 4 and 6 animals each with 2 and 1 animals, respectively, testing positive; +, denotes number of animals that tested positive.

In total, 152 animals comprising of eight different species were sampled in both areas. These comprised of 79 in Laikipia and 73 in Maasai Mara. All the animals responded well to the immobilisation drugs and induction times ranged between 8 and 12 min. No complications were encountered during immobilisation and handling except for a few animals, which had slightly elevated body temperatures that were attributed to physical exertion during darting as well as psychological stress and fear. These animals were cooled by applying copious amounts of water on the whole body.

**FIGURE 2:**
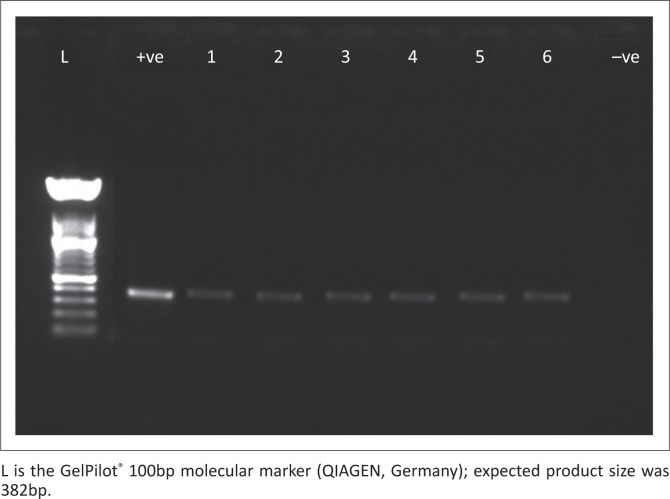
Gel image of polymerase chain reaction amplifications of the *gltA* gene.

Two of the 79 (2.5%) animals in Laikipia were found infected with SFG rickettsioses. Infections were found in a zebra (*Equus burchellii*) and a buffalo (*Syncerus caffer*) representing a prevalence of 2.6% and 3.2% in these species, respectively. Four of the 73 (5.5%) animals in Maasai Mara were infected with SFG rickettsioses. Infections were found in 1 of the 35 wildebeests (*Connochaetes taurinus*) and 3 of the 30 Topi (*Damaliscus lunatus* ssp. *jimela*) representing a prevalence of 2.9% and 30%, respectively. Infections were detected by amplification of the intergenic spacer *rpmE-*tRNA^fMet^ and *gltA* gene. These findings are summarised in Table 3. A representative gel image of PCR amplification of *gltA* gene is shown in Figure 2.

**TABLE 3:** Animals species sampled and prevalence of spotted fever group rickettsioses.

Animal species	Number positive (%) by amplification of the 2 genes				
	Number	gltA		rpmE-tRNA^fMet^	
		*n*	%	*n*	%
**Laikipia**					
Zebra (*Equus burchellii*)	39	1	2.6	1	2.6
Buffalo (*Syncerus caffer*)	31	1	3.2	1	3.2
Grant’s gazelle (*Nanger granti*)	7	0	0.0	0	0.0
Common waterbuck (*Kobus ellipsiprymnus* ssp. *ellipsiprymnus*)	2	0	0.0	0	0.0
**Total**	**79**	**2**	**2.5**	**2**	**2.5**
**Maasai Mara**					
Zebra (*Equus burchellii*)	21	0	0.0	0	0.0
Impala (*Aepyceros melampus*)	2	0	0.0	0	0.0
Wildebeest (*Connochaetes taurinus*)	35	1	2.9	1	2.9
Topi (*Damaliscus lunatus* ssp. *jimela*)	10	3	3.0	3	30
Coke’s hartebeest (*Alcelaphus buselaphus*)	5	0	0.0	0	0.0
**Total**	**73**	**4**	**5.5**	**4**	**5.5**

### Identification of *Rickettsia* spp.

**FIGURE 3:**
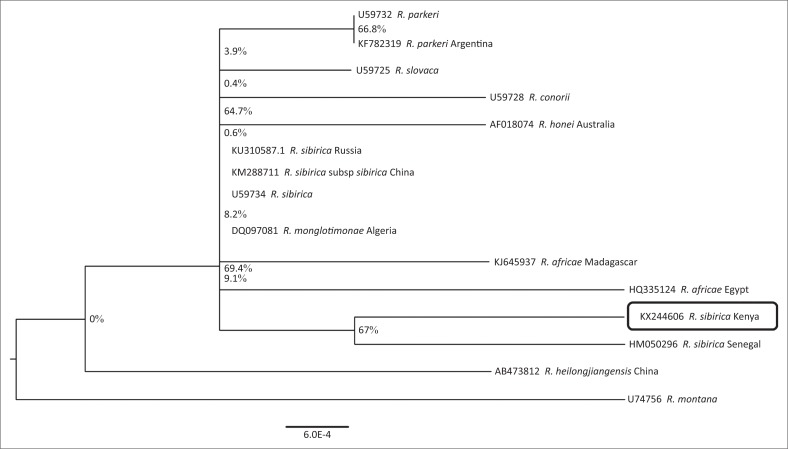
A phylogenetic relationship between *Rickettsia sibirica* isolated from Topi in Kenya and other Rickettsia sequences in the GenBank.

*Rickettsia sibirica* was identified in one sample obtained from a Topi (*Damaliscus lunatus* ssp. *jimela*) in Maasai Mara National Reserve. The isolate yielded a partial *gltA* sequence of 779 bp that had 99% identity to *R. sibirica* isolated from China Accession number KM288711 in the GenBank. The study sequence was submitted to the GenBank and provided accession number KX244606. The maximum likelihood phylogeny tree drawn using the study sequence and those similar to it in GenBank is shown in Figure 3. From the tree, the study sequence has a close relationship with other sequences from the same species in GenBank, such as *R. sibirica* from Senegal, while having a more distant relationship with the same species from other continents.

## Discussion

SFG rickettsioses can potentially be a public health concern in areas such as Laikipia and Maasai Mara which have unique human–livestock–wildlife interfaces that can potentially facilitate transmission of zoonotic infectious pathogens across different species. SFG rickettsioses were detected in 2.5% and 5.5% of wildlife sampled in Laikipia and Maasai Mara, respectively. This is the first report of the presence of the diseases in wildlife in Kenya, which demonstrates that wildlife may play a role in their spread. The finding of the presence of SFG rickettsioses in wildlife is consistent with a study by Zhang, Fan and Bi (1995) who, using PCR reported a prevalence of 7.4% in wild mice in China. Inokuma et al. (2008) and Ortuno et al. (2007) also reported the presence of SFG rickettsioses using PCR in a deer in Japan and a wild boar in Spain, respectively. Other studies by Boretti et al. (2009) and Barandika et al. (2007) using similar methods reported no detection in wild foxes in Switzerland and wild small mammals in Spain. The finding of low prevalence in wildlife is also comparable to a study in domestic animals by Maina (2012) who reported a prevalence of 3.7% in dogs and 7.7% in cats in western Kenya and no detection in cattle, sheep and goats. It is also comparable to a study by Kleinerman et al. (2013) who reported a prevalence of 2.0% in camels but no detection in horses in Israel. The finding, however, contrasts several other studies that have reported higher prevalence in domestic animals in Kenya. Using PCR, Mutai et al. (2013) reported a higher prevalence of 16.3% in cattle and 15.1% in sheep but a lower prevalence of 7.1% in goats from various parts of Kenya. Likewise, Kamani et al. (2015) reported a higher prevalence of 18.8% in camels in Nigeria.

The presence of *R. sibirica* has not been reported before in Kenya. The pathogen is widely distributed in North Asia (Jensenius, Fournier & Raoult 2004) with no reports available about its detection in Africa. It is the causative agent of North Asian tick typhus also called Siberian tick typhus (Jensenius, Fournier & Raoult 2004). The illness is characterised by fever, malaise, headache, myalgias and regional lymphadenopathy (Jensenius, Fournier & Raoult 2004; Ramos et al. 2013), which may be confused with those of other febrile infections leading to misdiagnosis. It is therefore of interest to understand how local populations in Laikipia and Maasai Mara cope with infections by *R. sibirica*.

The study documents the presence of SFG rickettsioses in wildlife, which suggests that wildlife can play a role in the epidemiology of the diseases. The finding underscores the risks for zoonotic transmission of SFG rickettsioses to humans and domestic animals at the wildlife–livestock interfaces in Laikipia and Maasai Mara. It is recommended that serological and molecular studies be initiated to determine SFG rickettsioses prevalence in local residents.
